# Supplementary material for: Development and Psychometric Properties of MisoQuest—A New Self-Report Questionnaire for Misophonia
Source: Int J Environ Res Public Health. 2020 Mar 10;17(5):1797. doi: 10.3390/ijerph17051797 (PMC7084437; doi:10.3390/ijerph17051797)
Supplement: Supplementary file 1 [file ijerph-17-01797-s001.zip › ijerph-730303-SI/ijerph-730303/file 3/60_items.docx]

1. I find clicking sounds (e.g. typing on a keyboard, clicking with a pen) unbearable.
2. I can’t control my anger when I hear someone eat.
3. I am bothered by some quiet noises made by people, but I don’t have a problem with louder ones.
4. I find some sounds made by the human body unbearable.
5. I find sounds made by my neighbours on the other side of the wall unbearable.
6. Most loud sounds cause me to experience unpleasant emotions*.*
7. I find sounds made by mouths (e.g. munching, crunching, smacking) unbearable*.*
8. I am bothered by some quiet noises, but I don’t have a problem with louder ones*.*
9. Sounds made by items such as phone ringtones, toilets flushing, refrigerator buzzing, cause me to experience very unpleasant emotions*.*
10. I find street sounds unbearable.
11. When I hear an unpleasant sound, I start to feel angry after several minutes.
12. I find some quiet sounds unbearable*.*
13. I find sounds made by things/items unbearable.
14. Sounds made by animals make me feel very unpleasant emotions*.*
15. When I hear some sounds, I am immediately overcome by unpleasant emotions*.*
16. When I hear an unpleasant sound, I immediately start to feel angry.
17. Unpleasant sounds have a negative influence on me only if they are prolonged*.*
18. Some unpleasant sounds make me feel angry immediately*.*
19. When I hear some unpleasant sounds, I start feeling emotions immediately*.*
20. Some sounds immediately irritate me.
21. I become nervous only after a sound does not stop for some time.
22. I start feeling anxious the moment I see a thing/animal/person that might make an unpleasant sound at any time.
23. I start feeling anger the moment I see a thing/animal/person who might make an unpleasant sound at any time.
24. When I know that I am about to hear an unpleasant sound, I start to get nervous.
25. When I hear an unpleasant sound, my heart starts to beat faster.
26. When I hear unpleasant sounds, I start sensing emotions in my body (e.g. I sweat, feel pain, feel pressure, my muscles tense).
27. When I hear some unpleasant sounds, I feel disgust.
28. When I hear some unpleasant sounds, I feel anxious.
29. Unpleasant sounds make me feel overwhelmed.
30. When I hear some unpleasant sounds, I feel anger.
31. I become anxious at the mere thought of an unpleasant sound.
32. Some sounds bother me so much that I have difficulty controlling my emotions.
33. I put a lot of effort into controlling emotions when I hear an unpleasant sound.
34. When I hear an unpleasant sound, I am scared that I will lose control over myself.
35. When I hear unpleasant sounds, my eyes start filling with tears.
36. When I hear unpleasant sounds, I get angry.
37. When I was a child, I shouted at people who made unpleasant noises.
38. As a child I would hit people who made unpleasant sounds.
39. I think that my reactions to sounds are stronger than those of other people.
40. I think that “something’s wrong with me” because I react too strongly to certain sounds.
41. When I hear unpleasant sounds, I feel that my emotions are too strong but I am unable to control them.
42. I believe that my reactions to sounds are too strong, but I can’t get rid of them.
43. I believe that my anger towards sounds is excessive.
44. Other people don’t experience reactions to sounds as strong and unpleasant as I do.
45. I leave the room if I hear an unpleasant sound.
46. I try to avoid situations in which unpleasant sounds may occur.
47. If I can, I avoid meeting with certain people because of the sounds they make.
48. I feel that my mental state worsens if I cannot leave a place where there’s an unpleasant sound..
49. I often think about how to drown out unpleasant sounds.
50. I plan my time in such a way that I have the least possible contact with unpleasant sounds..
51. I often think about unpleasant sounds.
52. I avoid family gatherings because of unpleasant sounds.
53. I sometimes forgo meeting with people I like in order to avoid unpleasant sounds.
54. Unpleasant sounds negatively impact my relationships with other people.
55. I am scared that unpleasant sounds may impact my future.
56. When meeting with other people, I am sometimes irritated because of unpleasant sounds that are present.
57. Unpleasant sounds make me unable to focus on a conversation.
58. Unpleasant sounds make me unable to focus on work.
59. I find it difficult to do everyday tasks because of unpleasant sounds.
60. My problem with sounds has a negative impact on my mental health.
