# Supplementary material for: Development and Psychometric Properties of MisoQuest—A New Self-Report Questionnaire for Misophonia
Source: Int J Environ Res Public Health. 2020 Mar 10;17(5):1797. doi: 10.3390/ijerph17051797 (PMC7084437; doi:10.3390/ijerph17051797)
Supplement: Supplementary file 1 [file ijerph-17-01797-s001.zip › ijerph-730303-SI/ijerph-730303/file 2/questionnaire_MisoQuest_ENG_PL.doc]

MisoQuest - kwestionariusz do pomiaru obniżonej tolerancji na dźwięki

*autorzy: Siepsiak, M., Śliwerski, A., Dragan, W. Ł*

Ludzie są mniej lub bardziej wrażliwi na pewne dźwięki. Czy istnieją dźwięki/odgłosy, które są dla Ciebie wyjątkowo uciążliwe? **Zaznacz, jak bardzo zgadzasz się lub nie zgadzasz z podanymi stwierdzeniami, według poniższego wzoru.**

1 - zdecydowanie nie zgadzam się

2 - nie zgadzam się

3 - trudno powiedzieć

4 - zgadzam się

5 - zdecydowanie zgadzam się

| 1 | Pewne dźwięki przeszkadzają mi tak bardzo, że mam trudność z opanowaniem emocji. | 1 | 2 | 3 | 4 | 5 |
| --- | --- | --- | --- | --- | --- | --- |
| 2 | Nieprzyjemne dźwięki sprawiają, że czuję się bezradna/ny. | 1 | 2 | 3 | 4 | 5 |
| 3 | Na samą myśl o nieprzyjemnym dźwięku robię się niespokojny/na. | 1 | 2 | 3 | 4 | 5 |
| 4 | Uważam, że moje reakcje na dźwięki są wyolbrzymione, ale nie mogę się ich pozbyć | 1 | 2 | 3 | 4 | 5 |
| 5 | Kiedy słyszę nieprzyjemne dźwięki, zaczynam odczuwać emocje w moim ciele (np. pocę się, czuję ból, czuję ucisk, napinają mi się mięśnie). | 1 | 2 | 3 | 4 | 5 |
| 6 | Na widok rzeczy/zwierzęcia/osób, które mogą zaraz wydać nieprzyjemny dźwięk, zaczynam odczuwać złość. | 1 | 2 | 3 | 4 | 5 |
| 7 | Wkładam dużo wysiłku w opanowanie emocji, kiedy słyszę nieprzyjemny dźwięk. | 1 | 2 | 3 | 4 | 5 |
| 8 | Jeśli mogę, to unikam spotkań z pewnymi ludźmi, ze względu na wydawane przez nich dźwięki. | 1 | 2 | 3 | 4 | 5 |
| 9 | Pewne dźwięki wydawane przez ciało ludzkie są dla mnie nie do wytrzymania. | 1 | 2 | 3 | 4 | 5 |
| 10 | Czuję, że mój stan psychiczny pogarsza się, jeśli nie mogę opuścić miejsca w którym jest nieprzyjemny dźwięk. | 1 | 2 | 3 | 4 | 5 |
| 11 | Często myślę o tym, jak zagłuszyć nieprzyjemne dźwięki. | 1 | 2 | 3 | 4 | 5 |
| 12 | Pewne nieprzyjemne dźwięki sprawiają, że natychmiast zaczynam odczuwać złość. | 1 | 2 | 3 | 4 | 5 |
| 13 | Boję się, że nieprzyjemne dźwięki mogą mieć wpływ na moją przyszłość. | 1 | 2 | 3 | 4 | 5 |
| 14 | Zdarza się, że podczas spotkań z innymi ludźmi jestem rozdrażniony/na z powodu obecnych tam nieprzyjemnych dźwięków. | 1 | 2 | 3 | 4 | 5 |

MisoQuest – a questionnaire for assessing decreased sound tolerance

*authors: Siepsiak, M., Śliwerski, A., Dragan, W. Ł*

Some people are less sensitive to certain sounds, while other people are more sensitive to certain sounds. Are there any sounds which you find particularly burdensome? Please indicate how much you agree or disagree with the following statements using the following scale:

1 – I definitely do not agree

2 – I do not agree

3 – Hard to say

4 – I agree

5 – I definitely agree

| 1 | Some sounds bother me so much that I have difficulty controlling my emotions. | 1 | 2 | 3 | 4 | 5 |
| --- | --- | --- | --- | --- | --- | --- |
| 2 | Unpleasant sounds make me feel overwhelmed. | 1 | 2 | 3 | 4 | 5 |
| 3 | I become anxious at the mere thought of an unpleasant sound. | 1 | 2 | 3 | 4 | 5 |
| 4 | I believe that my reactions to sounds are exaggerated, but I can’t get rid of them. | 1 | 2 | 3 | 4 | 5 |
| 5 | When I hear unpleasant sounds, I start sensing emotions in my body (e.g. I sweat, feel pain, feel pressure, my muscles tens). | 1 | 2 | 3 | 4 | 5 |
| 6 | I start feeling anger the moment I see a thing/animal/person that might make an unpleasant sound at any time. | 1 | 2 | 3 | 4 | 5 |
| 7 | I put a lot of effort into controlling emotions when I hear an unpleasant sound. | 1 | 2 | 3 | 4 | 5 |
| 8 | If I can, I avoid meeting with certain people because of the sounds they make. | 1 | 2 | 3 | 4 | 5 |
| 9 | I find some sounds made by the human body unbearable. | 1 | 2 | 3 | 4 | 5 |
| 10 | I feel that my mental state worsens if I cannot leave a place where there’s an unpleasant sound. | 1 | 2 | 3 | 4 | 5 |
| 11 | I often think about how to drown out unpleasant sounds. | 1 | 2 | 3 | 4 | 5 |
| 12 | Some unpleasant sounds make me instantly angry. | 1 | 2 | 3 | 4 | 5 |
| 13 | I am scared that unpleasant sounds may impact my future. | 1 | 2 | 3 | 4 | 5 |
| 14 | When meeting with other people, I am sometimes irritated because of unpleasant sounds that are present. | 1 | 2 | 3 | 4 | 5 |
